# Learning curve of percutaneous endoscopic interlaminar lumbar discectomy versus open lumbar microdiscectomy at the L5–S1 level

**DOI:** 10.1371/journal.pone.0236296

**Published:** 2020-07-30

**Authors:** Seong Son, Yong Ahn, Sang Gu Lee, Woo Kyung Kim

**Affiliations:** Department of Neurosurgery, Gil Medical Center, Gachon University College of Medicine, Incheon, South Korea; George Washington University, UNITED STATES

## Abstract

**Objective:**

Many studies on the clinical outcome of full endoscopic spine surgery versus open spine surgery have been published. However, only a few studies have compared the learning curves of percutaneous endoscopic interlaminar lumbar discectomy (PEILD) and open lumbar microdiscectomy (OLM) at the L5–S1 level. This study included patients with disc herniation at the L5–S1 level, who underwent PEILD or OLM performed by a single novice surgeon and compared the learning curves.

**Methods:**

Fifty-six patients who underwent PEILD or OLM at the L5–S1 level and completed a minimum 1-year follow-up were enrolled in the study. The patients were allocated to the PEILD group (n = 27, September 2014 to August 2016) or an OLM group (n = 29, September 2012 to August 2014). The learning curves were retrospectively compared based on operation time and surgical outcomes, including complication, failure, and recurrence rates were retrospectively compared.

**Results:**

Significant intergroup differences were not noted with respect to the baseline characteristics, including age, sex, body mass index, preoperative symptoms, or preoperative radiological findings. The mean operation time was significantly shorter in the PEILD group than in the OLM group (63.89±17.99 min versus 78.03±19.01 min, p = 0.006). Based on the operation time according to the number of cases, the learning curve was more difficult in the PEILD group according to the cumulative analysis (case number cut-off for proficiency was 18 in the PEILD group versus 10 in the OLM group) and linear regression analysis (proportionality constant for decrease in the operation time was -0.922 in the PEILD group versus -1.738 in the OLM group) than that in the OLM group. However, the surgical outcomes, including failure, surgical efficacy based on nerve root decompression, complication, and recurrence rates did not differ between the two groups.

**Conclusion:**

Although the learning curve of PEILD was more difficult than that of OLM, the mean operation time was shorter in the PEILD group than that in the OLM group. Moreover, based on the surgical outcomes, PEILD showed efficacy and safety similar to those of OLM.

## Introduction

Open lumbar microdiscectomy (OLM) has been a standard surgical technique for the treatment of lumbar disc herniation owing to its efficacy and safety, since the late 1907s [1–6]. In contrast, percutaneous endoscopic interlaminar lumbar discectomy (PEILD) has gained popularity worldwide, as a standard full-endoscopic surgical technique for the treatment of disc herniation at the L5–S1 level and as an alternative minimally invasive technique to OLM since the mid-2000s [7]. Recently, many previous studies have reported on the efficacy and safety of PEILD [8–14].

Many recent comparative studies have insisted that there is no significant difference in the clinical results between full endoscopic surgery and OLM; they also claimed that full endoscopic surgery is better than OLM as it is a minimally invasive surgery [15–23]. However, there is a definite threshold skill for a surgical technique that involves a surgical approach using a single percutaneous small port and an endoscope for obtaining a magnified view of the unfamiliar two-dimensional narrow surgical space. The major concerns regarding full endoscopic surgery include a steep learning curve, fear of failure, complications, and recurrence after surgery, particularly if the surgeon is in the novice stage.

There is no methodical study comparing the learning curves of PEILD and OLM, based on the operation time and surgical outcomes, including failure, surgical efficacy, complication, and recurrence rates, for the treatment of lumbar disc herniation at the L5–S1 level performed by a single surgeon with novice level proficiency. To the best of our knowledge, this is the first retrospective matched cohort study to report on this comparison.

## Materials and methods

### Surgical indications and patient population

The study was approved by the Institutional Review Board of our institute (GFIRB2020-105). The ethics committee waived the requirement of obtaining informed consent and all data were fully anonymized before we accessed them.

A single surgeon performed spine surgeries in a single institute from September 2012 onwards after the surgeon had completed 1.5 years of a fellowship. At first, the surgeon performed only OLM in patients with disc herniation at the L5–S1 level till August 2014. From September 2014 onwards, the surgeon performed PEILD in patients with soft disc herniation at the L5–S1 level after completing a 4-week fellowship and extensive cadaveric training for full endoscopic surgery. Consequently, the surgeon’s proficiency in OLM and PEILD was similar to that of a novice during the first 2 years of practice.

The indications for lumbar discectomy surgery were, as follows: 1) persistent low back pain and radiating leg pain despite administration of adequate (at least 6 weeks) conservative treatment, 2) severe pain affecting the ability to perform daily life activities, or 3) severe paresis (motor grade 3 or less).

A retrospective chart review was performed for all patients who underwent OLM (between September 2012 and August 2014) or PEILD (between September 2014 and August 2016) at the L5–S1 level. To minimize the effect of multi-level or bilateral surgery on the outcomes, we identified patients who underwent single-level unilateral discectomy at the L5–S1 level and were followed-up for at least 12 months.

During the study period, 40 and 31 patients underwent single–level unilateral OLM and PEILD, respectively. Therefore, the average term between two cases was not significantly different the two groups (0.60 month in the OLM group versus 0.77 month in the PEILD group).

To avoid selection bias due to different characteristics of disc herniation between the two groups, patients were excluded base on the following criteria: 1) hard (calcified) disc herniation detected on preoperative magnetic resonance imaging (MRI) or computed tomography (CT), 2) foraminal or extraforaminal disc herniation, 3) upward migrated disc fragments, 4) previous history of lumbar spine surgery, and 5) an inability to complete the preoperative and postoperative questionnaires or missing medical records.

**Fig 1:**
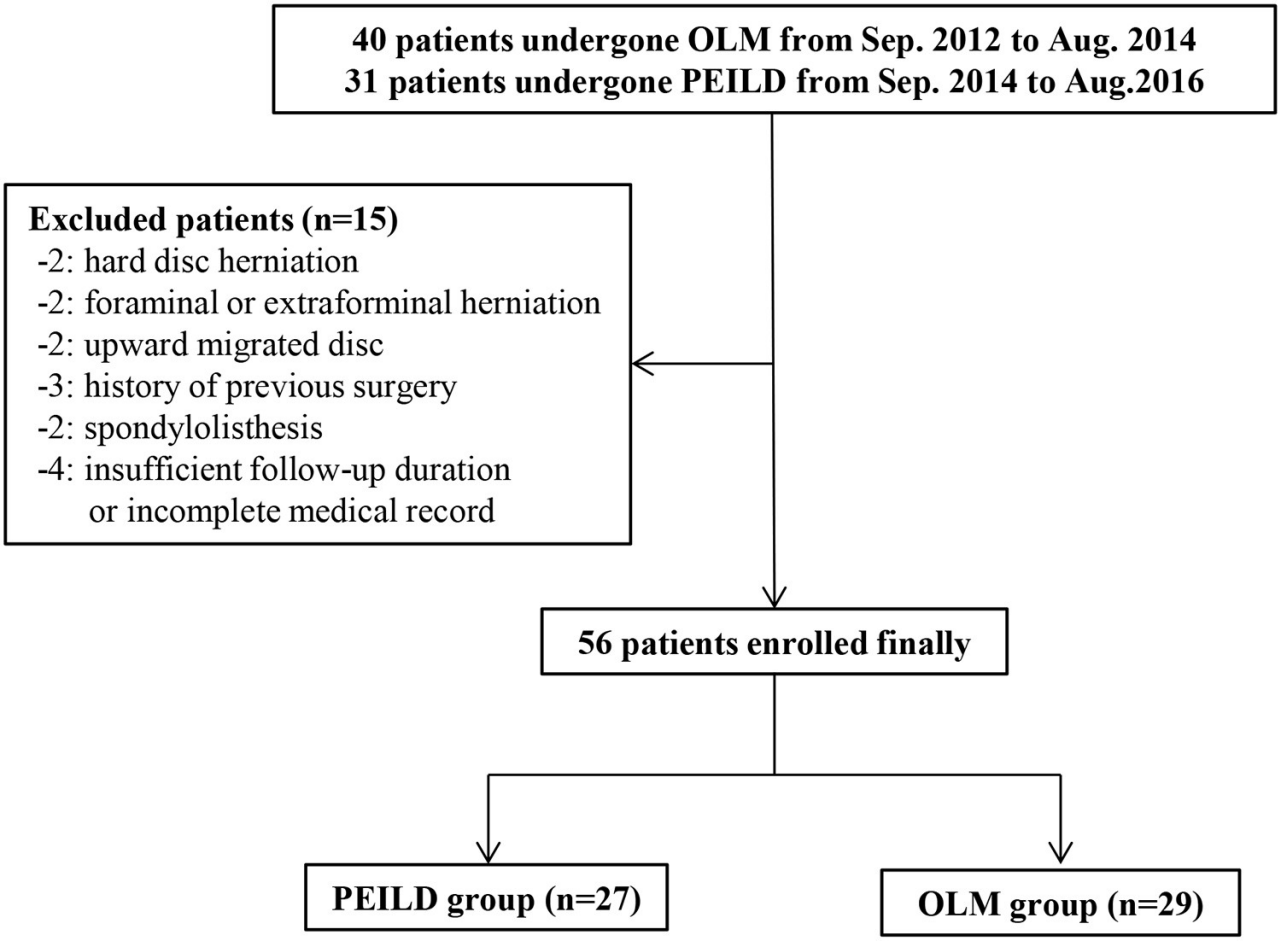
Patient selection process. OLM, open lumbar discectomy; PEILD, percutaneous endoscopic interlaminar lumbar discectomy.

After excluding 15 patients among the 71 patients, the remaining 56 patients were enrolled in the final study cohort, with 27 patients in the PEILD group and 29 patients in the OLM group (Fig 1).

### Operative technique

All surgical procedures were performed by a single surgeon. After induction of general anesthesia, all patients were positioned in the prone position with decreased abdominal pressure.

In the PEILD group, a 0.5-cm paramedian skin incision was created 0.5–1.0cm away from the midline, according to the anatomical variations of the interlaminar space. After the insertion of an obturator into the laminar-inferior articular process junction, the working cannula and endoscope (Vertebris system, Richard Wolf, Knittlingen, Germany; Joimax system, Joimax, Irvine, CA, USA) were inserted. Under endoscopic guidance with continuous irrigation, the ligamentum flavum was punctured after partially removing and/or splitting it. The surgeon sometimes performed a laminotomy and/or medial facetectomy using a high-speed drill, according to the preoperative plan. The ruptured disc material was removed, and disc space evacuation was performed on a case by case basis; annuloplasty was performed using radiofrequency (Elliquence Int, Hewlett, NY, USA). Finally, the wound was closed using one- or two-point subcutaneous sutures and skin tape.

In the OLM group, a midline 2.5–3.0-cm skin incision was created, followed by periosteal dissection and application of a Caspar-type retractor. Under microscopic guidance, after partial laminotomy and/or medial facetectomy using a high-speed drill and removal of the ligamentum flavum using the Kerrison punch, decompression and disc space evacuation were performed, depending on the patient characteristics. Finally, the wound was closed using layer-by-layer sutures from the fascia to the skin with or without drain insertion, based on the extent of bleeding and the surgeon’s discretion.

In both groups, decisions regarding the equipment to be uses, bone work, intervertebral disc evacuation, and drain insertion were made, depending on the preoperative planning and/or intra-operative findings.

### Evaluation of learning curve based on the operation time

The demographic data and baseline characteristics, including age, sex, occupation, smoking status, alcohol consumption, body mass index, history of previous nerve block, trauma history, preoperative symptom duration, and presence of weakness, were analyzed.

Lumbar MRI was performed prior to the surgery in all patients. The preoperative status, including disc degeneration grade according to the Pfirrmann grade [17], rupture side (right or left), rupture type (disc level or migration), and ruptured disc volume, were evaluated. The ruptured disc volume was assessed as the volume index determined as the product of the largest width, length, and height of the ruptured disc fragment, as determined on MRI.

The total operation time was determined as the sum of preoperative preparation time, duration between skin incision and wound closure, and postoperative recovery duration. We assessed the learning curve by analyzing the operation time, defined as the duration between skin incision and skin closure. The operation time was recorded according to the case number, and cumulative analysis of the operation time was performed to determine a cut-off value of familiarity. Furthermore, linear regression analyses were performed to evaluate the linear correlation between the operation time and number of cases.

### Surgical outcome evaluation

**Fig 2:**
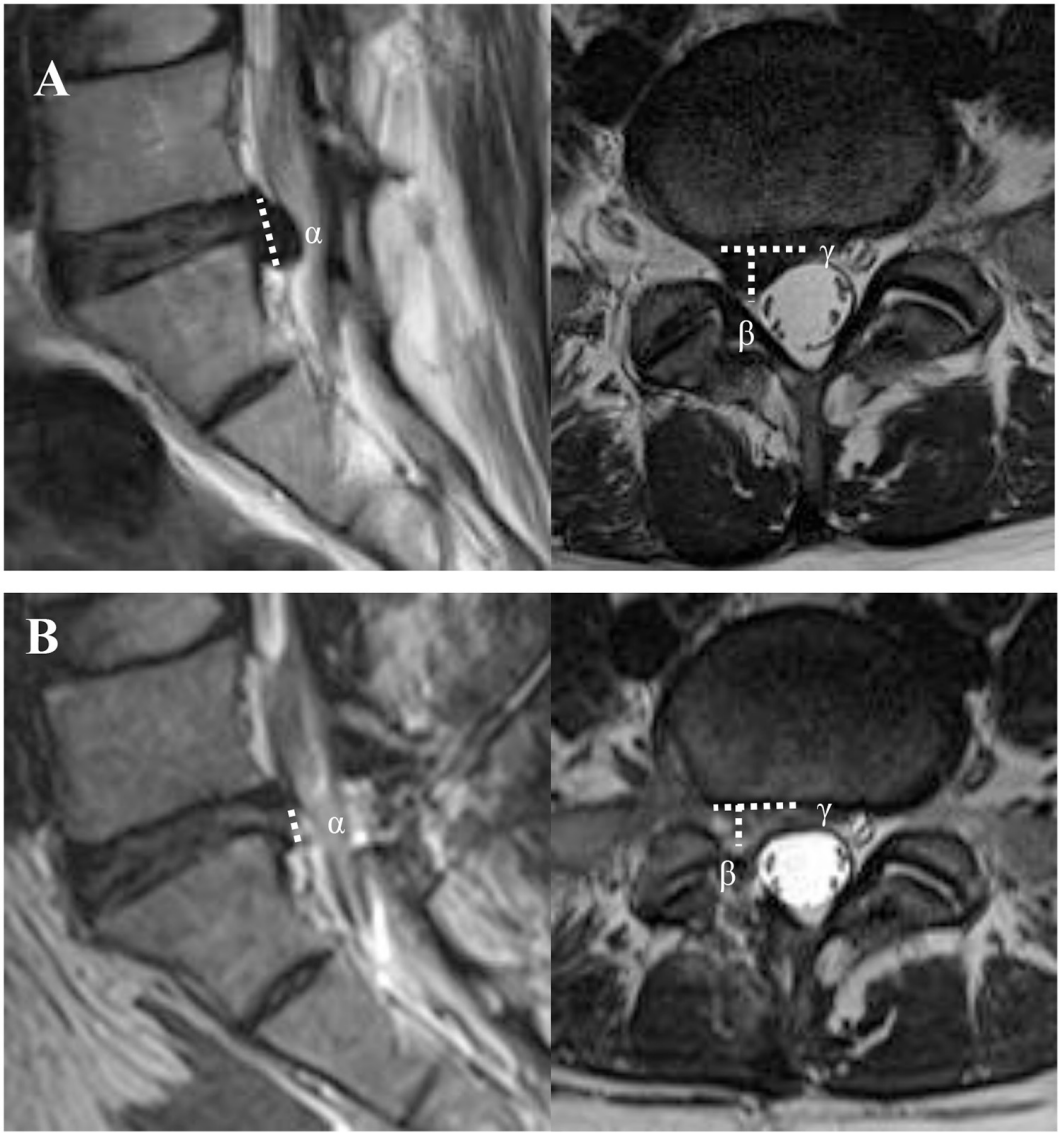
Assessment of the ruptured disc volume index (mm^3^) as the product of [height (α), length (β), and width (γ) recorded in (A) preoperative and (B) postoperative MRI scans. MRI: magnetic resonance imaging.

We evaluated the surgical outcomes, including intraoperative blood loss, surgical failure rate, remnant ruptured disc volume, complication rate, and recurrence rate. The intraoperative blood loss was indirectly evaluated using preoperative and postoperative hemoglobin levels. The surgical failure rate was defined as the conversion rate to OLM during or after PEILD. We performed MRI immediately postoperatively for all patients to confirm nerve root decompression and check for any postoperative complications, such as epidural hematoma. To assess the surgical efficacy, the remnant ruptured disc volume was evaluated from the immediate postoperative MRI data. The remnant ruptured disc volume was evaluated using the same volume index method as that used in the preoperative evaluation (Fig 2).

The surgical complications included durotomy during the surgery, symptomatic postoperative epidural hematoma, neurologic deterioration, surgical site infection, and perioperative morbidity, such as cardiopulmonary issues, deep vein thrombosis, and urinary complications. The recurrence rate and additional procedures, including revision surgery and nerve root block, were also investigated within 12 months postoperatively.

### Statistical analysis

Data management and statistical analysis were performed using SPSS version 23.0 (SPSS Inc., Chicago, IL, USA). The Pearson’s chi square test, paired samples t-test, independent t-test, and non-parametric Mann-Whitney U-test were used according to the characteristics of the data. Cumulative analysis and linear regression analysis were used to analyze the learning curve based on the operation time. On the other hand, Kaplan-Meier survival analysis was used to investigate survival without the need for additional procedures or revision surgery. The data are expressed as means ± standard deviations or mean and 95% confidence interval (CI), depending on the data distribution. *P* values < 0.05 were considered statistically significant.

## Results

### Demographic data and baseline characteristics

The study population comprised 31 men and 25 women with a mean age of 42.09±12.86 years, mean body mass index of 24.00±3.32, and mean symptom duration of 12.00 weeks (95% CI, 7.03–16.97).

A significant difference was not noted in the demographic data and symptom-related baseline characteristics between the PEILD and OLM groups. Moreover, intergroup differences were not noted in the preoperative MRI findings, including disc degeneration, ruptured disc characteristics, and ruptured disc volume (Table 1).

**Table 1:** Demographic data and baseline characteristics.

		PEILD (n = 27)	OLM (n = 29)	P value
**Age (years)**		42.85±14.84	41.38±10.92	0.673*
**Sex**				0.602†
	Male	16	15	
	Female	11	14	
**Occupation**				0.617†
	White collar	12	13	
	Blue collar	8	6	
	Others	7	10	
**Smoking status**		7	9	0.771†
**Alcohol consumption**		14	10	0.280†
**Height (cm)**		167.69±9.40	167.90±9.72	0.937*
**Weight (kg)**		65.67±9.48	69.83±14.57	0.215*
**Body mass index (kg/m**^**2**^**)**		23.35±2.92	24.61±3.60	0.161*
**Symptom duration (weeks)**		12.81 (95% CI, 5.93–19.70)	11.24 (95% CI, 3.68–18.81)	0.987‡
**Previous block**		12	13	0.977†
**Trauma**		2	1	0.511†
**Weakness**		8	5	0.273†
**Pfirmann grade**				0.596†
	III	20	18	
	IV	6	10	
	V	1	1	
**Side**				0.586†
	Right	12	15	
	Left	15	14	
**Type of ruptured disc**				0.643†
	Migrated	7	6	
	Subligamentous	20	23	
**Volume index of the ruptured disc (mm**^**3**^**)**		1222.74±597.25	1095.83±883.41	0.563*

CI, confidence interval; OLM, open lumbar microdiscectomy; PEILD, percutaneous endoscopic interlaminar lumbar discectomy.

*Independent t-test;

^†^Pearson’s Chi square test;

^‡^Nonparametric Mann-Whitney U-test.

### Learning curve for PEILD and OLM based on operation time

The preoperative preparation time and time required for postoperative recovery time from anesthesia did not differ between the two groups. However, the operation time was significantly shorter in the PEILD group than in the OLM group (63.89±17.99 min versus 78.03±19.01 min; p = 0.006 using the independent t-test). Moreover, the total operation time determined as the sum of preoperative preparation time, operation time, and postoperative recovery times was shorter in the PEILD group than in the OLM group (141.22±29.17 min versus 159.41±25.11 min; respectively, p = 0.015 using the independent t-test) (Table 2).

**Table 2:** Operation time.

	PEILD (n = 27)	OLM (n = 29)	P value
**Preoperative preparation time (min)**	36.48 (95% CI, 30.38–42.59)	35.17 (95% CI, 32.19–38.16)	0.596*
**Operation time (min)**	63.89±17.99	78.03±19.01	0.006†
**Postoperative recovery time (min)**	40.48 (95% CI, 31.09–49.87)	46.21 (95% CI, 42.28–50.13)	0.518*
**Total operation time (min)**	141.22±29.17	159.41±25.11	0.015†

CI, confidence interval; OLM, open lumbar microdiscectomy; PEILD, percutaneous endoscopic interlaminar lumbar discectomy.

*Nonparametric Mann-Whitney U-test;

†Independent t-test.

**Fig 3:**
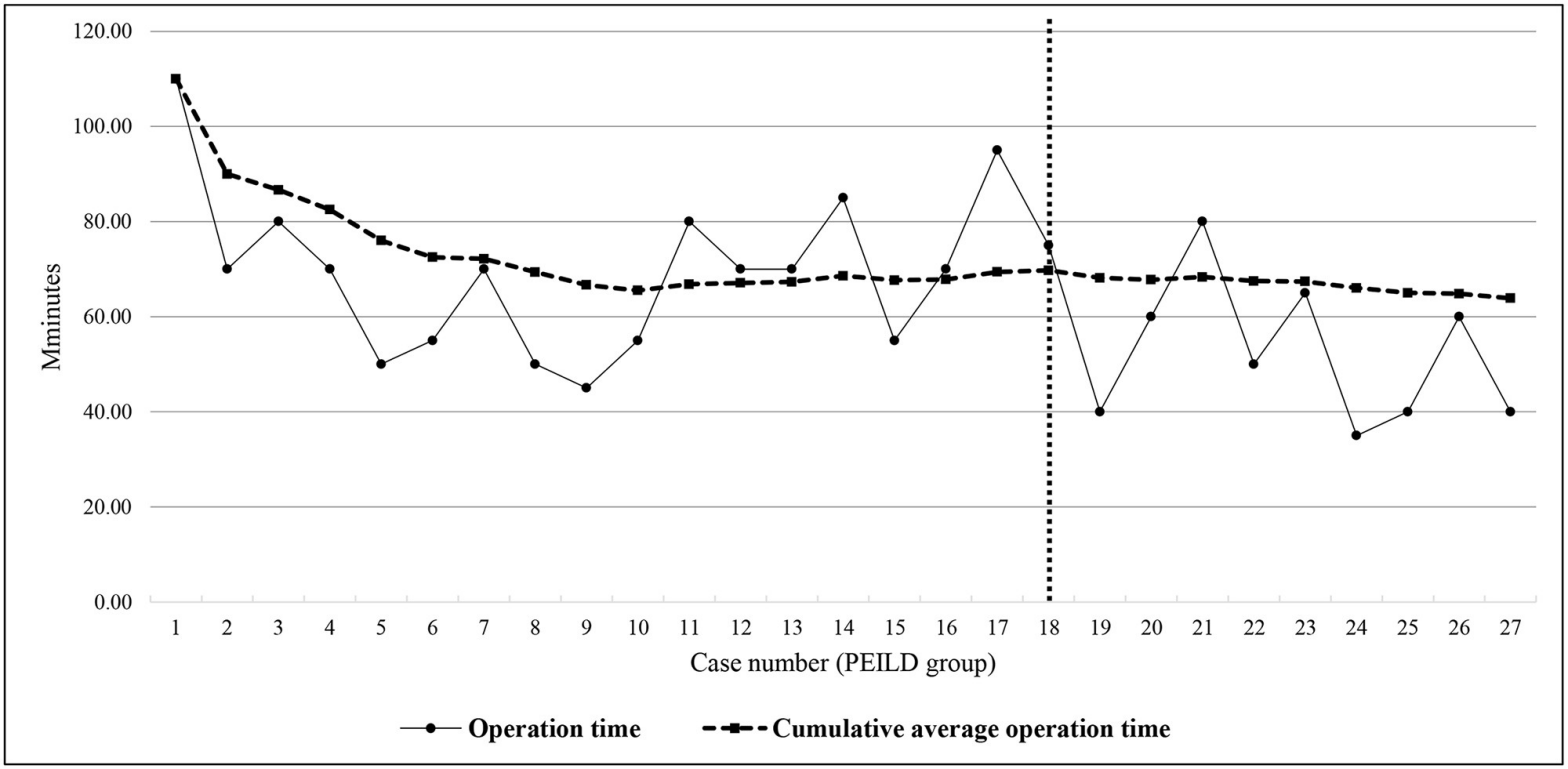
Operation time according to the number of cases and cumulative mean operation time in the percutaneous endoscopic interlaminar discectomy (PEILD) group.

According to the cumulative analysis, the operation time decreased with the accumulation of cases, and the cumulative average operation time was consistent after 18 cases in the PEILD group. The mean operation time was most significantly different between the first 18 cases and the last 9 cases (69.72±16.76 min versus 52.22±15.02 min, respectively; p = 0.014 using the independent t-test) indicating that the cut-off for proficiency was 18 cases in the PEILD group (Fig 3).

**Fig 4:**
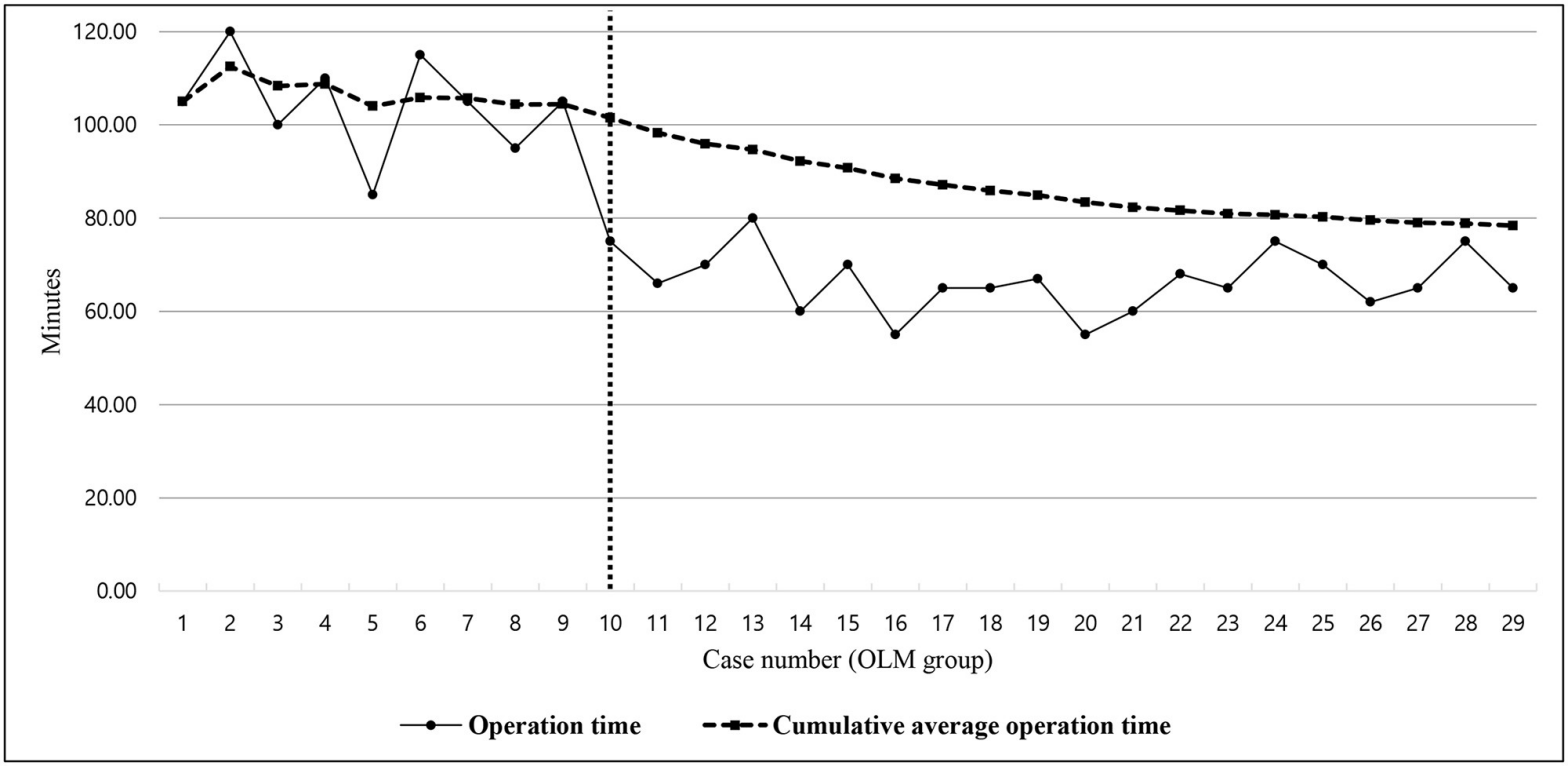
Operation time according to the number of cases and cumulative mean operation time in the open lumbar microdiscectomy (OLM) group.

On the other hand, the operation time decreased with the accumulation of cases and the cumulative average operation time appeared to saturate after 10 cases in the OLM group. The mean operation time was most significantly different between the first 10 cases and the last 19 cases (101.50±13.55 min versus 66.21±6.43 min, respectively; p<0.001 using the independent t-test) indicating that the cut-off for proficiency was 10 cases in the OLM group (Fig 4).

**Fig 5:**
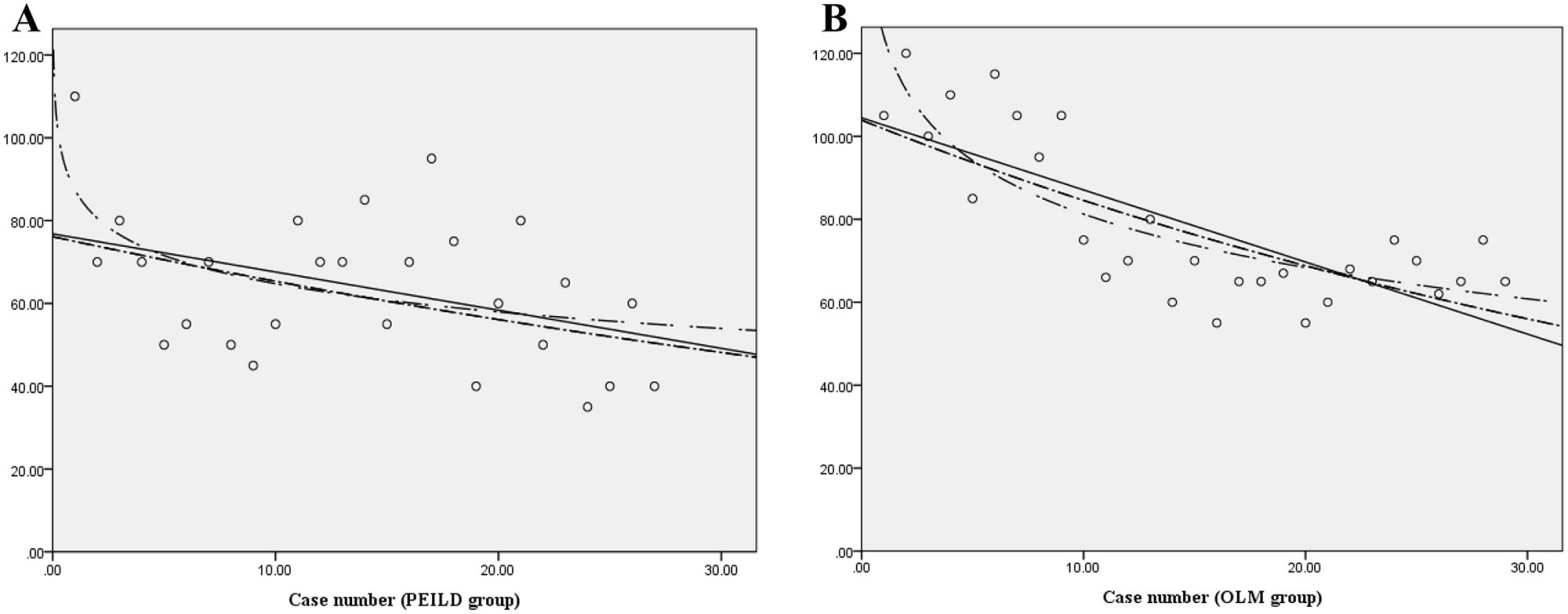
Linear regression analysis for the percutaneous endoscopic interlaminar discectomy (PEILD) group and the open lumbar microdiscectomy (OLM) group. A. Operation time of PEILD. B. Operation time of OLM. CI, confidence interval.

According to the linear regression analysis, the operation time in the PEILD group was calculated using the following formula: operation time = 76.795 (95% CI, 63.129–90.461) − 0.922 (95% CI, -1.775 to -0.069) × case number (p = 0.035). This suggests a significant decrease in the operation time with the degree of inclination of -0.9222 based on the number of operations performed. Conversely, in the OLM group, the operation time was determined using the following formula: operation time = 104.448 (95% CI, 94.434–114.462) − 1.738 (95% CI, -2.321 to -0.155) × case number (p<0.001). This suggests a significant decrease in the operation time with the degree of inclination of -1.738 based on the number of operations performed (Fig 5).

In summary, although the initial and mean operation times were shorter in the PEILD group, a larger number of cases was required to achieve proficiency in the PEILD group, and the inclination for the reduced operation time was less steeper in the PEILD group.

### Surgical outcome

During the surgical procedure, bone work using high-speed drill and drain insertion were significantly more commonly performed in the OLM group (93.1% cases of bone work, 48.3% cases of drain insertion) than in the PEILD group (14.8% cases of bone work, 0% cases of drain insertion) (p<0.001 using Pearson’s Chi square test). However, the preoperative and postoperative hemoglobin levels, indicative of intraoperative blood loss, did not differ significantly between the two groups (Table 3).

**Table 3:** Surgical outcomes.

	PEILD (n = 27)	OLM (n = 29)	P value
**Bone work**	4 (14.8%)	27 (93.1%)	<0.001†
**Disc space evacuation**	23 (85.2%)	24 (82.8%)	0.805†
**Drain insertion**	0	14 (48.3%)	<0.001†
**Preoperative hemoglobin (g/dL)**	14.03±1.47	14.30±1.33	0.500*
**Postoperative hemoglobin (g/dL)**	13.78±1.72	13.79±1.44	0.978*
**Postoperative volume index of remnant disc (mm**^**3**^**)**	164.52±89.56	122.45±69.17	0.315*
**Surgical failure**	0	0	NA
**Complication**	2 (7.4%)	2 (6.9%)	0.596†t3fb
**Recurrence**	2 (7.4%)	3 (10.3%)	0.941†
**Revision surgery**	2 (7.4%)	2 (6.9%)	0.700†
**Additional nerve block**	1 (3.7%)	0	0.296†

NA, not available; OLM, open lumbar microdiscectomy; PEILD, percutaneous endoscopic interlaminar lumbar discectomy.

* Independent t-test;

†Pearson’s Chi square test.

In both groups, there were no cases of failure, that is conversion to OLM during PEILD, or significant nerve root decompression failure. We did not note a significant intergroup difference in the frequency of disc space evacuation and remnant ruptured disc volume, suggesting a similar degree of nerve root decompression and surgical efficacy, between the two groups.

The complication rate did not differ between the two groups. In the PEILD group, incidental intraoperative tiny durotomy occurred in two patients (7.4%). In contrast, surgical complications developed in 2 patients (6.9%) in the OLM group; incidental durotomy occurred intraoperatively in one patient and transient voiding difficulty occurred in one patient postoperatively. However, severe complications, such as surgical site infection and permanent postoperative neurologic deficit, were not noted in either group (Table 3).

**Fig 6:**
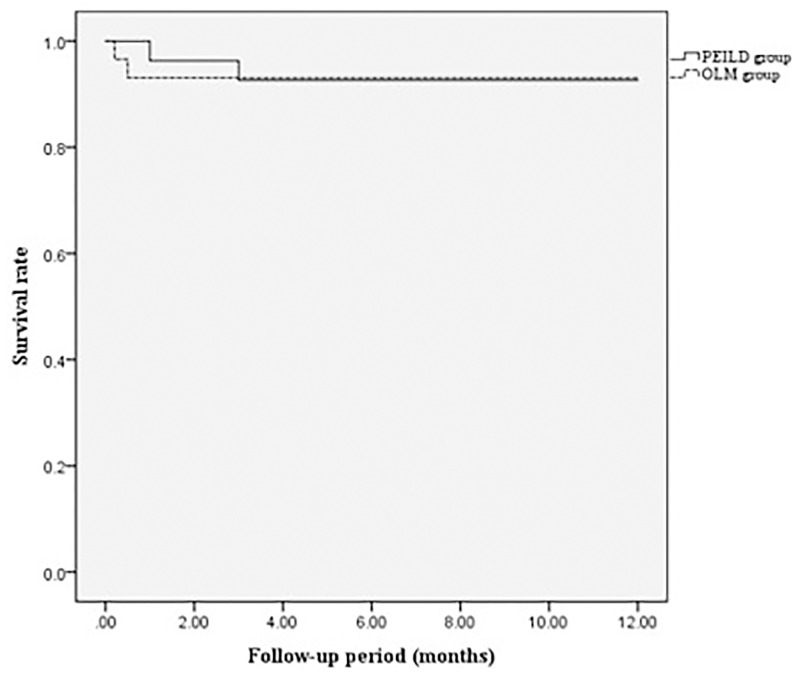
Survival rate according to the Kaplan-Meier survival analysis between the two groups (p = 0.986 using log rank [Mantel-Cox] test). OLM, open lumbar discectomy; PEILD, percutaneous endoscopic interlaminar lumbar discectomy.

The recurrence, additional nerve block, and revision surgery rates were not different between the two groups. In the PEILD group, two patients (7.4%) experienced recurrence of disc herniation and underwent revision surgery (one patient at 1.3 months and the other patient at 5 months postoperatively), and one patient underwent an additional nerve block procedure for symptom control at 4 months postoperatively due to recurrent leg pain. In the OLM group, three patients (10.3%) experienced recurrence of disc herniation and underwent revision surgery (two patients within 10 days, and one patient at 1 month postoperatively); however, additional nerve blocks were not noted (Table 3). The Kaplan-Meier survival analysis revealed that the survival rate did not differ between the two groups during the follow-up period (p = 0.986 using log rank [Mantel-Cox] test; Fig 6).

## Discussion

The operation time is a major parameter in the evaluation of a surgeon’s technical competence [24]. Incompetence is inevitable while learning a new surgical technique, particularly a minimally invasive surgical approach; thus, sufficient training is mandatory for novices until an acceptable performance level is achieved. The surgeon’s comfort and technical proficiency are correlated to a decrease in the procedure duration in a chronological case series, and the learning curve has been traditionally evaluated based on the operation time according to the number of cases [25].

In our study, the overall mean operation time (63.89±17.99 min in the PEILD group versus 78.03±19.01 min in the OLM group, p = 0.006 using the independent t-test) and the mean operation time at the proficient stage (52.22±15.02 min in the last 9 cases in the PEILD group versus 66.21±6.43 min in the last 19 cases in the OLM group, p = 0.002 using the independent t-test) were shorter in the PEILD group than in the OLM group. Additional surgical procedures, such as layer-by-layer opening and closure, higher incidence of bone work in laminotomy, and higher incidence of drain insertion in the OLM group may lead to a longer operation time even at the proficient stage.

As the number of surgical procedures performed increased, the operation time decreased owing to familiarity with the surgical technique in both groups. Considering a consistent operation time in both groups, the cumulative analysis revealed a difference in the proficiency threshold between the two groups (18 cases in the PEILD group versus 10 cases in the OLM group). In the PEILD group, the mean operation time decreased from 69.72±16.76 min to 52.22±15.02 min (25.1% decrease) and approached an asymptote after the 18^th^ case, while the mean operation time in the OLM group decreased from 101.50±13.55 min to 66.21±6.43 min (34.8% reduction) and approached an asymptote after the 10^th^ case. Moreover, the linear regression analysis showed a more effective and favorable degree of inclination for operation time reduction in the OLM group than in the PEILD group (proportionality constant of -0.922 in the PEILD group versus -1.738 in the OLM group). In summary, the basis of the cut-off number of cases for achieving proficiency and degree of inclination for the operation time reduction revealed that the learning curve for the PEILD group was more difficult than that for the OLM group.

Compared with the previous studies, the learning curve of PEILD at the L5–S1 level is reasonable. Based on the asymptote point, our results showed that the learning curve of PEILD is similar to that for the initial 10–30 cases of other minimally invasive spinal surgeries, such as microsurgery using a tubular retractor or other full endoscopic spine surgery [26–29]. Based on the 25.1% decrease in the operation time and proportionality constant of -0.922 in the formula for the operation time, the rate of decline in the PEILD group is not difficult compared with the 23–58% decrease in the operation time during the initial 10–30^th^ cases in the previous reports [26–29]. This finding implies that the barriers encountered by novices during the beginning phase of learning to perform PEILD are similar to those noted during the beginning phase of learning to perform other minimally invasive spine surgeries.

Another clinically relevant parameter used to assess a surgeon’s proficiency is the surgical failure or complication rate. The lack of clear anatomic knowledge or orientation and unfamiliarity with new instruments appears to be significant limitations and may lead to serious neurologic injury or unintended adverse events during surgery [30]. Previous study showed that surgical failure and complication were usually encountered in the novice stage of the learning process [25]. Moreover, several studies on minimally invasive spinal surgery have reported a higher complication rate and poorer clinical outcome at the novice level than at the proficient level [20, 26, 31, 32].

However, in our study, the surgical outcomes, including intraoperative blood loss, surgical failure rate, surgical success of nerve root decompression according to immediate postoperative MRI, complication rate, and recurrence rate, were similar between the two groups. In addition, the complication rate of 7.4% in the PEILD group was favorable compared with the complication rate of 14–40% previously reported for other minimally invasive spinal surgeries performed by surgeons at the novice stage [25, 30, 33, 34]. Furthermore, there were no severe complications such as permanent neurologic deficits or surgical site infection and no cases of surgery-related morbidity. These findings suggest that PEILD at the L5–S1 level is safe and relatively easy to learn with fewer complications compared with other minimally invasive spinal surgeries.

This study has some limitations. First, due to its retrospective nature, it was impossible to control for all variations. Nevertheless, we tried to minimize errors by precluding variables associated with the results. Although a single surgeon at the similar novice stage may not have completely same proficiency in OLM and PELID, we could reduce the bias due to involving multiple surgeons in this study. Second, the number of patients in the final cohort and the follow-up period were insufficient. However, to the best of our knowledge, this study is the first study to compare the learning curves of the PEILD and OLM at the L5–S1 level with the same proficiency and same indication. More complete studies with a large number of patients or a prospective study is required to validate our results.

## Conclusion

Based on the operation time, the learning curve of PEILD was more difficult than that of OLM. However, the learning curve of PEILD was not as difficult as those of other minimally invasive spine surgeries, and the operation time of PEILD was significantly shorter than that of OLM. Moreover, the safety and efficacy of PEILD were similar to OLM with comparable failure, complication, and recurrence rates.
